# Asymptomatic foot and ankle structural injuries: a 3D imaging and finite element analysis of elite fencers

**DOI:** 10.1186/s13102-022-00444-y

**Published:** 2022-03-27

**Authors:** Congfei Lu, Yuxuan Fan, Genyu Yu, Hua Chen, Jonathan Sinclair, Yifang Fan

**Affiliations:** 1 Foot Research Laboratory, Key Laboratory of Sport and Health Science of Fujian Province, School of Physical Education and Sport Science, Fujian Normal University, Fuzhou, 350117 China; 2 Research Centre for Applied Sport, Physical Activity and Performance, School of Sport and Health Sciences, University of Central Lancashire, Lancashire, Preston, PR1 2HE UK

**Keywords:** Sesamoid, First metatarsophalangeal joint, Trail foot, Lunge, Elite fencers, Biomechanical monitoring

## Abstract

**Background:**

Fencing is a highly asymmetrical combat sport, that imposes high mechanical demands over repeated exposures on the musculoskeletal structures, a primary cause of injuries in fencers. However, there are limited epidemiological studies on the structural injuries of the foot and ankle in fencers. This study aimed to investigate foot and ankle structural injuries, and explore how metatarsophalangeal joint structural changes may affect the mechanisms of foot and ankle injuries in asymptomatic fencers.

**Methods:**

3D images of foot and ankle morphology using computed tomography were obtained from ten elite fencers. We then constructed finite element models of the first metatarsophalangeal joint in the foot of their trail legs. The validated models were used to simulate stress distribution changes from different ankle joint angles during lunging.

**Results:**

The findings showed that stress distribution changes at the medial and lateral sesamoid may have caused sesamoid fractures, and that habitual and concentrated stress on the metatarsal bones might have flattened the sesamoid groove. This process may damage the integrity of the first metatarsophalangeal joint, and consequently affect the efficiency of the windlass mechanism in fencers. During lunging, different ankle joint angles of the trail foot increased the total stress difference of the medial and lateral foot, and thus influenced the lunging quality and its stability.

**Conclusions:**

Our findings revealed that the asymmetric nature of fencing might have caused asymptomatic foot and ankle structural injuries, and finite element analysis results indicated that this might increase the incidence of the serious injuries if unattended. Regular computed tomography examination should be introduced to monitor elite fencers’ lower limb alterations, permitting unique angle adjustments in the trail foot without sacrificing technical or physiologic properties based on the exam results and reduce the lower limb injury risk.

## Background

Fencing is regarded as a highly asymmetrical combat sport that requires a dominant side (fencing side) of body for sparring and competition. Owing to its asymmetrical nature, fencing imposes high mechanical demands over repeated exposures on the musculoskeletal structures, which is recognised as the primary cause of injuries in fencers [1]. Previous analyses have shown that the majority of fencing injuries are chronic in nature [2], and experienced primarily in the lower extremities [3]; although there is only limited epidemiological information concerning structural injuries (SI) of the foot and ankle [2].

SI at the foot and ankle are classified as avulsion fractures, stress fractures, and joint deformities [4, 5]. However, whilst epidemiological analyses have shown that lower limb tendon structural alterations are common in asymptomatic fencers [5], most fencers were importantly unaware that they had suffered from a foot and ankle SI. Existing studies have shown that serious SIs in fencing competitions are relatively uncommon, with 184 time-loss injuries reported during a 5-year study of US national competitions, representing an overall rate of 0.3 per 1000 athlete exposures [2]. The results of a prospective cohort investigation of university fencers found that the injury rate in 1 year was 0.243%, of which ankle injuries accounted for 25% of all injuries [6]. A study on the risk of time-loss injuries found that the injury rate of 85,686 participants was 0.028%, of which ankle sprain was the most common one, with an incidence rate of 25.3% [7]. However, taking into account the experimental approaches adopted by previous epidemiological analyses using interview and questionnaire-based techniques [2, 6], it can be postulated that the current epidemiological literature base in fencing is insufficient [6]. Structural damages are typically assessed using X-ray [8], but existing methods have not yielded reliable conclusions because two-thirds of magnetic resonance imaging, computed tomography (CT), nuclear medicine images were not appropriately diagnosed [9], and because patients are not able to remain in the same position at over multiple examinations [10]. Three-dimensional (3D) reconstruction of CT scan can help observe the asymptomatic SI of the foot and ankle by rotation, zooming, transparency or by virtually removing some bony elements [11].

Finite element analysis (FEA) of human body structures represents a widely adopted approach in biomechanical studies, as it can derive indices of mechanical loading at complex musculoskeletal structures during human movement using computer processing techniques [12]. Li et al. developed and validated a specific finite element model of foot designed to compare and study the stress level and stress increase rate of the metatarsals of different foot strike modalities during running [13]. Their findings showed that when performing the same movement, different loading conditions increased the strain level of metatarsals for 20–30% [13]. FEA findings also showed that the geometric changes caused variations in metatarsal peak value of stress [14, 15]. These studies demonstrate that FEA can examine the impact of injury on the athlete's loading condition and offer a reference for monitoring increase/decreases in injury risks.

The lunge is recognised as the main attacking mechanism in competitive fencing; in which the athlete aligns the lead foot with their opponent whilst maintaining trail foot angulation to maintain a stable guard position [16]. Studies in performance analysis have shown that male fencers lunge once every 23.9 s during fencing competitions, which has attracted much attention from researchers [16, 17]. The first metatarsophalangeal joint (MTPJ) of the trail foot is vital during movement in fencers, and almost all fencing footwork movements are initiated at the first MTPJ; indicating that this joint and its surrounding structures may be at high risk of chronic pathologies [12]. During lunging, the ankle rotation of the trail foot affects the average lunge velocity and strength [18]. This foot displacement changes the direction of the ankle joint force. Therefore, finite element analytical modelling of the first MTPJ of the trail foot during the fencing lunge may provide clinically meaningful information regarding the biomechanical mechanisms of asymptomatic foot and ankle SI induced by common fencing movements. However, there is currently no existing finite element model capable of quantifying stress loading of the first MTPJ during the fencing lunge.

Our findings of asymptomatic SI from elite fencers’ CT images led us to construct three FEA models for asymptomatic injuries of distinct seriousness to assess how each one influenced the elite fencers’ loading conditions, and whether the asymptomatic injuries might add the risk of other more serious pathologies.

## Methods

### Ethics

This study was approved by the Ethics Committee of Fujian Normal University (FJNU20210059). All experiments were performed in accordance with the university’s set guidelines and regulations. All participants signed an informed consent form to take part in this study.

### Participants

This study recruited 10 elite fencers (Age: 23.1 ± 3.36 years, Height: 183.2 ± 6.49 cm, Weight: 75.1 ± 10.76 kg, Competitive experience: 13.70 ± 3.29 years) from the Provincial Men's Fencing Team. Table 1 shows the general information of the participants.

**Table 1:** Basic information of the participants

Fencers	Fencing weapons	Age	Years of experience in fencing competition	Height (cm)	Mass (kg)	Dominant hand
P1	Foil	22	12	178	68	Right
P2	Foil	28	17	185	72	Right
P3	Epee	18	8	178	64	Right
P4	Foil	23	13	180	67	Left
P5	Epee	24	15	197	103	Right
P6	Foil	26	18	184	75	Left
P7	Foil	23	13	175	75	Right
P8	Foil	17	9	191	75	Right
P9	Foil	23	14	178	68	Right
P10	Epee	27	18	186	84	Left

### Procedure

This study was divided into two parts—imaging diagnosis and FEA.

CT images (0.5 mm slices) of both ankles were collected without loading in the anteroposterior position from each participant using a CT scanner (Toshiba/Aquilion ONE, Toshiba, Japan). We selected CT images from three participants (P2, P3 and P7) with different asymptomatic injuries, conducted FEA and validated it by collecting their barefoot data during lunging on the Zebris FDM gait analysis system.

### CT scan and 3D reconstruction of CT scan

**Fig. 1:**
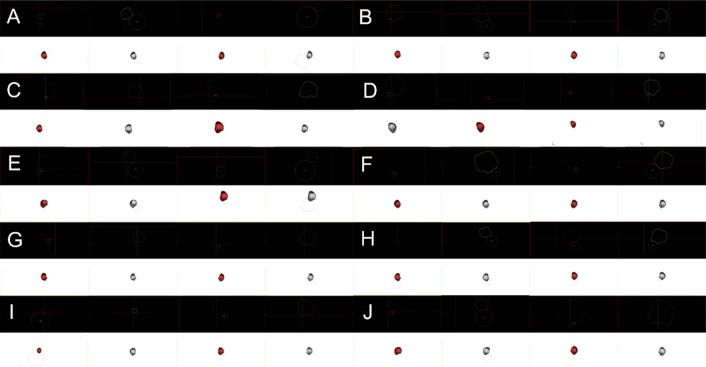
Fractures of participants’ first metatarsal medial and lateral grooves of sesamoids. **A**–**J** are fencers P1–P10, respectively

The scan results were stored as DICOM files. To increase resolution and precision, different fields of view of the left and right feet were exported. A 3D foot arch with standardized coordinate system was constructed in *Mimics* (Mimics Research 17.0 for X64, Materialize, Leuven Belgium) [19]. CT imaging was used to screen for participants’ ankle SI and the results were recorded. Mimics software used volume rendering to reproduce the foot and ankle of fencers during scanning. Thresholding, region growing, and morphology operations were used to reconstruct the tibia, fibula, tarsus, metatarsals, and sesamoids of participants in Mimics. A previously described method [20] was used to measure the curvature diameter of the sesamoid groove (SG) of the first metatarsal bone (Fig. 1).

We screened the imaging results, and selected the 3D reconstructed finite element model consisting of the first MTPJ, sesamoid, and medial cuneiform of the trail foot (left foot) of Participant 2 (P2), 3 (P3), and 7 (P7), as P2 had a lateral sesamoid fracture, P3 had a medial sesamoid fracture, and P7 had an intact MTPJ and sesamoid.

### Finite element attributes

#### Geometric model

**Fig. 2:**
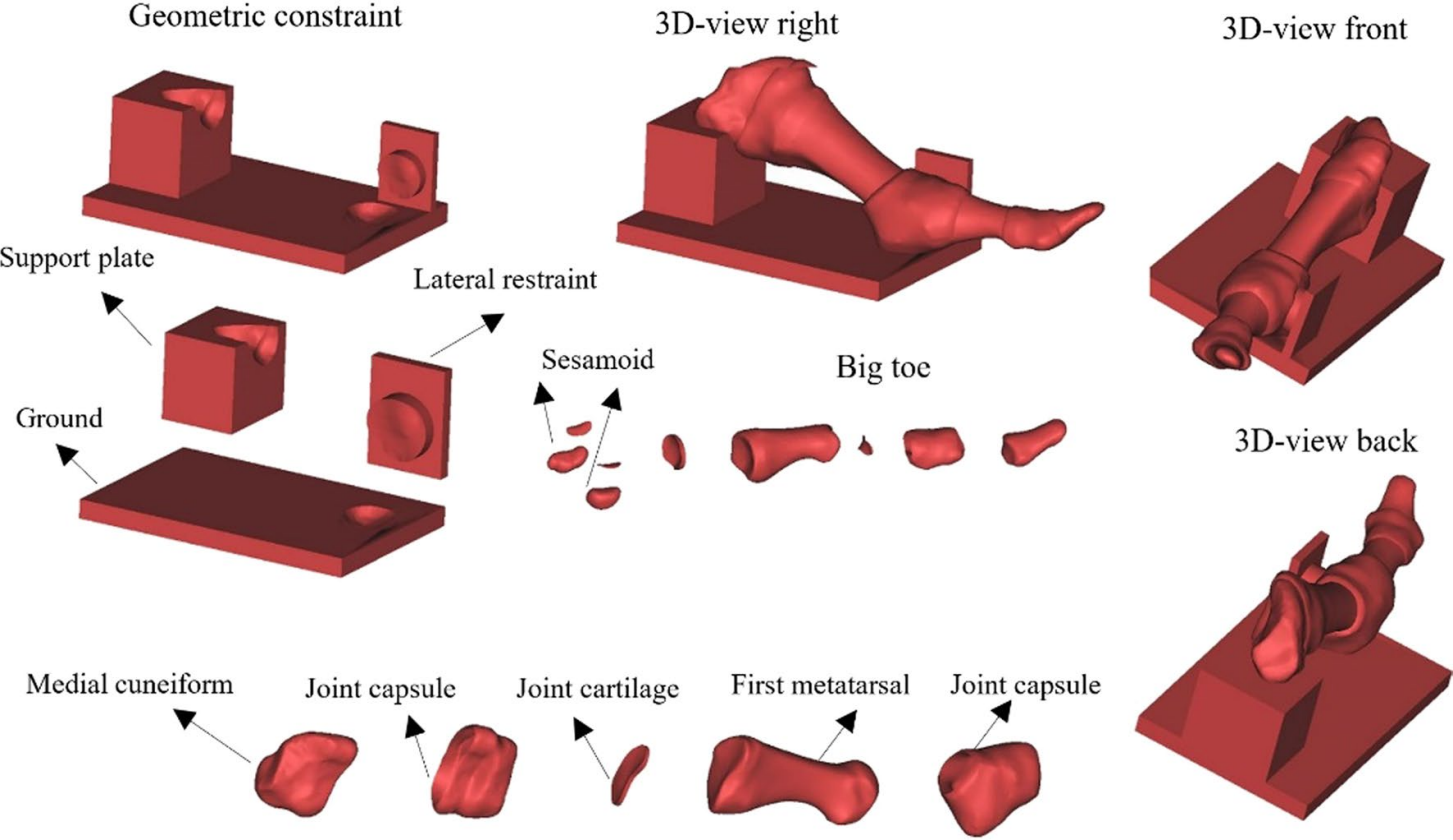
Composition of the finite element model. The joint capsule simulated the constraint of the ligament and soft tissue. The lateral constraint simulated the constraints of the other metatarsal bones. The support platform simulated the support of the other components in the medial arch on the MTPJ. The ground simulated the support provided by the ground on the MTPJ

The finite element model and model composition was shown in Fig. 2.

#### Mesh and material attributes

To simplify the problem, the MTPJ model was idealized as homogeneous, isotropic, and linearly elastic. In Ansys workbench (Ansys 14.5, Ansys Inc., Canonsburg, USA), automated manner was used to generate finite element meshes. The complete element mesh involves about 80,000 nodes and 40,000 elements. Table 2 shows the elements and material attributes [21]. The MTPJ structures of the three participants were used to simulate the stress impact experienced by the front sole of the trail foot during the lunge.

**Table 2:** Elements and material attributes

Component	Young’s modulus (mpa)	Poisson’s ratio	Element size (mm)	Element type
Foot bone (support plate)	7300	0.3	5	Tetrahedra
Joint cartilage	1	0.4	1	Tetrahedra
Joint capsule (lateral restraint)	300	0.4	5	Tetrahedra
Ground	30,000	0.3	5	Brick

#### Loads and boundary conditions

To examine the effects of ankle joint angle during lunges on the MTPJ [22], we simulated the loading on the MTPJ when the ankle joint angle ranged from 0° to 45° during lunging. In the sagittal plane, an angle of 90° was presented between the trail and lead foot; then, we set the force direction of the MTPJ as 0°. When the angle between the trail and lead foot was 45°, the force direction of the MTPJ was also 45°, i.e., the force direction of the MTPJ was the rotation angle of the ankle. Therefore, we simplified the support of the medial arch on the MTPJ as a rectangle and the compression of the first metatarsal bone by the other metatarsal bones was simplified as lateral constraints.

#### Basic outcome measures

We extracted the maximum principal stress (Max-PS), minimum principal stress (Min-PS) and total stress (TS) from the FEA results and calculated the average value and coefficient of variation (CV) of the stress difference (D-value) under the lateral and medial SG of the first metatarsal bone. Principal stress was considered as the gold standard to evaluate skeletal stress distribution. It was a normal stress when the shear stress was nil in a specific region, which was used to evaluate the yielding condition of uniaxial loading in the specific region [23]. TS referred to the total load in the specific region [24] to evaluate the load transmission of bone geometric form. CV indicated the variability of the metatarsal structure under different angles of force loading, and it reflected whether the metatarsal structure was affected by the angle of force loading. D-value indicated the Max-PS peak value difference between the lateral and medial sesamoid grooves, which was related to the sesamoid bone injuries.

#### Validation of the finite element model

We selected three participants to validate the finite element model as Table 3 shows that P2 and P3 had asymptomatic injuries of the left and right sesamoid bone of the first metatarsal in the trail foot, and P7 had no asymptomatic injury to the sesamoid bone in the trail foot. Each participant therefore represented a distinct injury modality, facilitating our comparative analysis. We conducted FEA and validated it by collecting P2, P3 and P7’s barefoot gait data during lunging on the Zebris FDM gait analysis system. They were asked to jog for five minutes and then to do five minutes of static stretching prior to the test. As the testing was undertaken barefoot, they were firstly required to use 75% alcohol to disinfect both feet. The initial testing procedures were demonstrated by the laboratory assistant. They stood on the force platform facing the target. Participants were required to lunge at their target with maximum effort and 3 trials were recorded with a rest period of 1 min after each. After 3 trials, they were informed of their correct landing position. Then, they stepped onto the gait analysis system (Zebris Medical GmbH, Isny, Germany − 0.56 (W) × 6.08 (L) m). The platform operated at a sampling frequency of 100 Hz, with a sensor density of 1.4 sensors/cm^2^. We retrieved the plantar pressure distribution diagram and the dynamic change process of the ground reaction force and compared the results using the finite element model.

**Table 3:** Participants’ foot and ankle structural injuries

	MS	LS	CU	MC	NA	CA	TA	FI	TI
P1									
Left		⊙						✓	
Right		⊙					✓	✓	
P2									
Left		✓				✓			
Right	✓	✓	✓		✓	✓	✓		
P3									
Left	✓								
Right									
P4									
Left							✓		
Right					✓		✓		
P5									
Left						✓		✓	
Right	✓					✓		✓	
P6									
Left									✓
Right	✓								✓
P7									
Left				✓	✓		✓		
Right							✓	✓	
P8									
Left	✓								✓
Right									✓
P9									
Left	✓								
Right									
P10									
Left			✓		✓		✓		✓
Right			✓		✓		✓		✓

Key: Left/Right, left and right ankle; *MS* medial sesamoid; *LS* lateral sesamoid; *CU* cuboid; *MC* medial cuneiform; *NA* navicular; *CA* calcaneus; *TA* talus, *FI* fibula; *TI* tibia. ✓ fracture; ⊙ absence

## Results

**Fig. 3:**
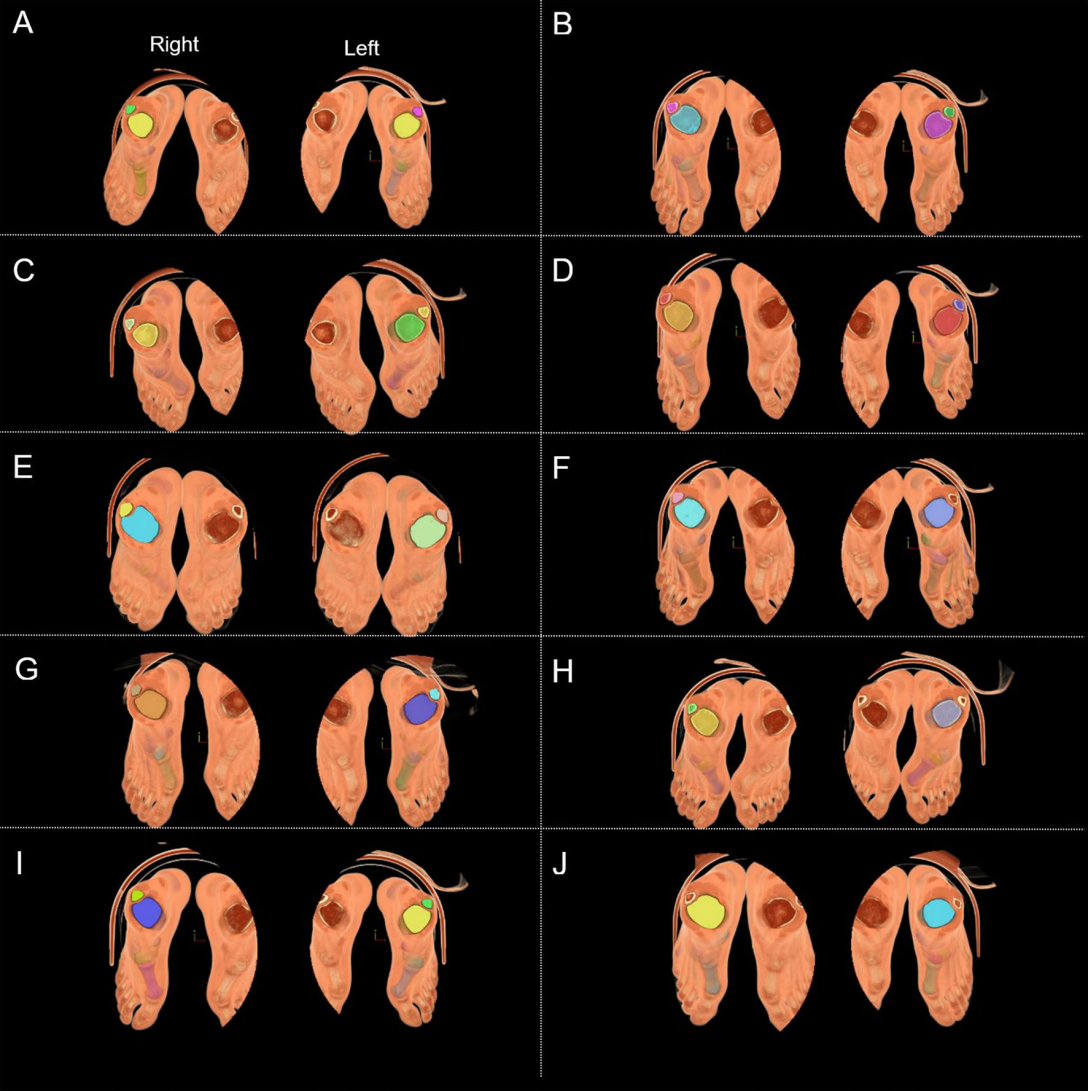
Participants’ CT scanned foot posture. **A**–**J** refer to P1–P10, respectively. To improve the resolution and voxel isotropy of the cross-sectional image [25], the fields of view of the left foot and right foot were set up and stored

The ankle posture and field size during CT scanning were compared (Fig. 3).

**Fig. 4:**
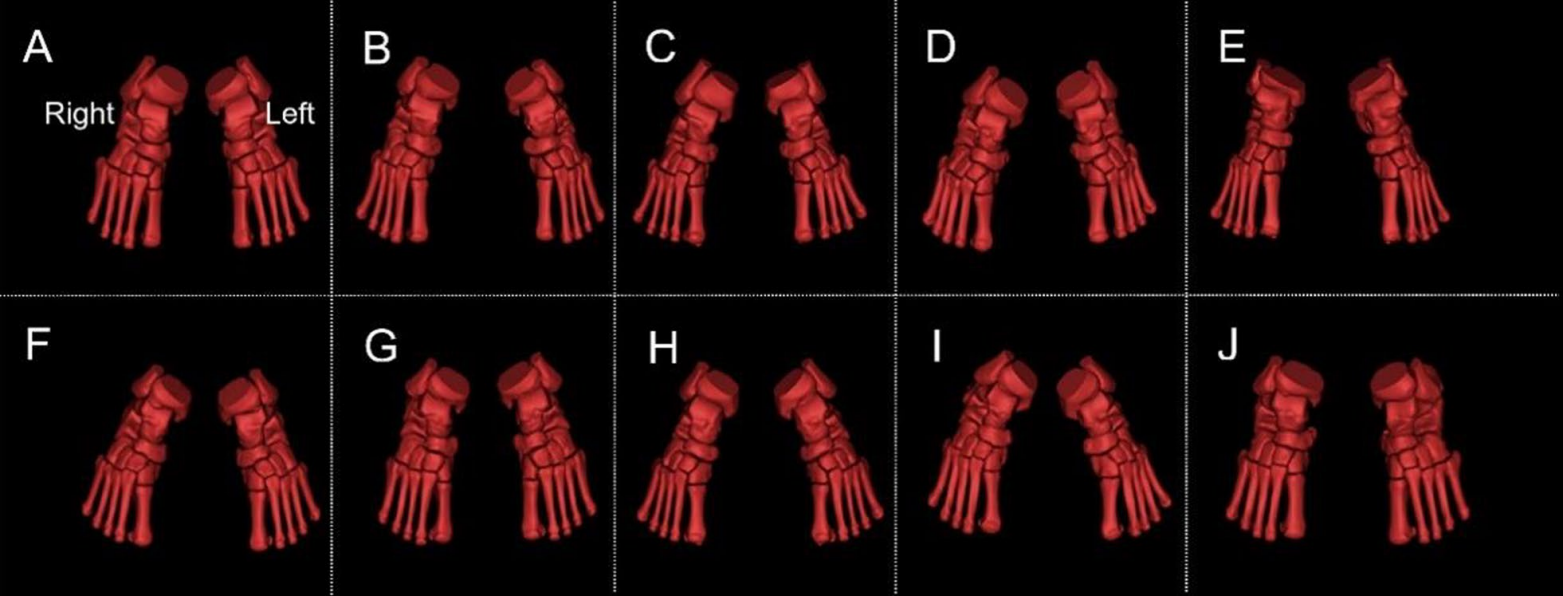
Participants’ tibia, fibula, tarsal, metatarsal, and sesamoid. **A**–**J** refer to P1–P10, respectively. As the postures of the participants during scanning were different, coordinate system of bones on the ankle was standardized. “Right” and “Left” refer to the right and left ankle structure, respectively

3D reconstructions of participants’ tibia, fibula, tarsal, metatarsal, and sesamoid were shown in Fig. 4.

**Fig. 5:**
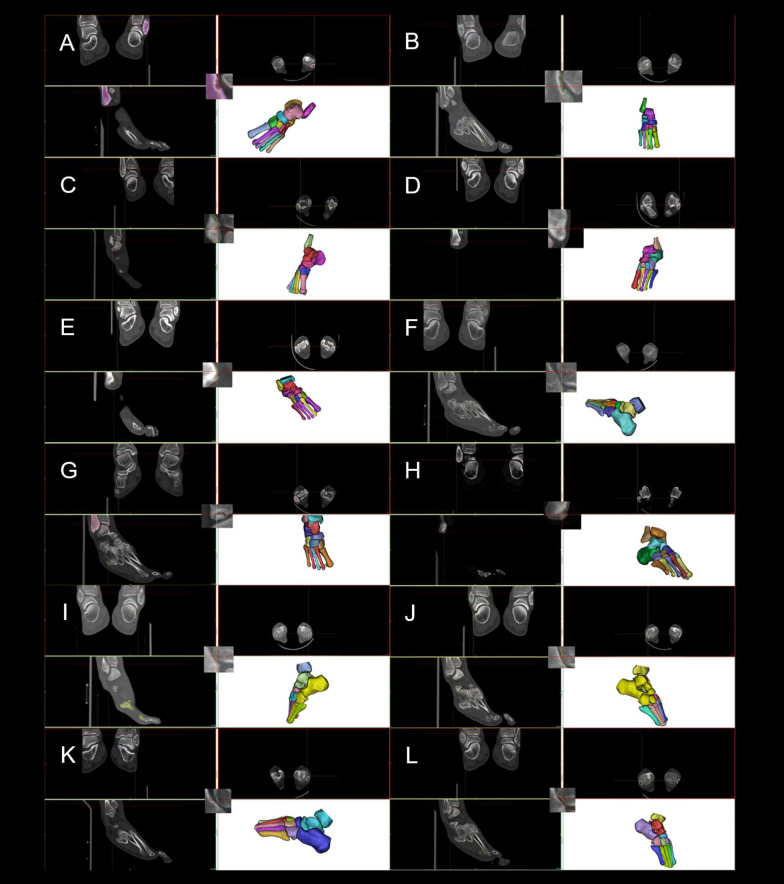
Fractures of participants’ fibula and tibia. **A** P1 left fibula. **B** P1 right fibula. **C** P3 right fibula. **D** P5 left fibula. **E** P5 right fibula. **F** P6 left tibia. **G** P6 right tibia. **H** P7 right fibula. **I** P8 left tibia. **J** P8 right tibia. **K** P10 left

**Fig. 6:**
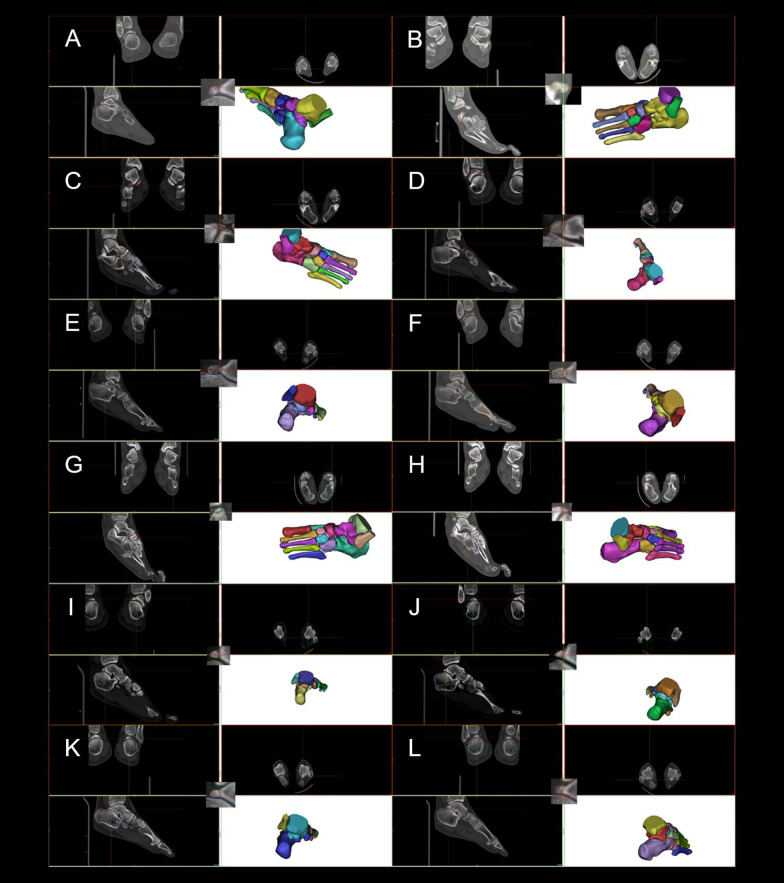
Fractures of participants’ calcaneus and talus. **A** P1 left talus. **B** P2 right calcaneus. **C** P2 left calcaneus. **D** P2 left talus. **E** P4 right talus. **F** P4 left talus. **G** P5 right calcaneus. **H** P5 right calcaneus. **I** P7 left talus. **J** P7 right talus. **K** P10 left talus. **L** P10 right talus

**Fig. 7:**
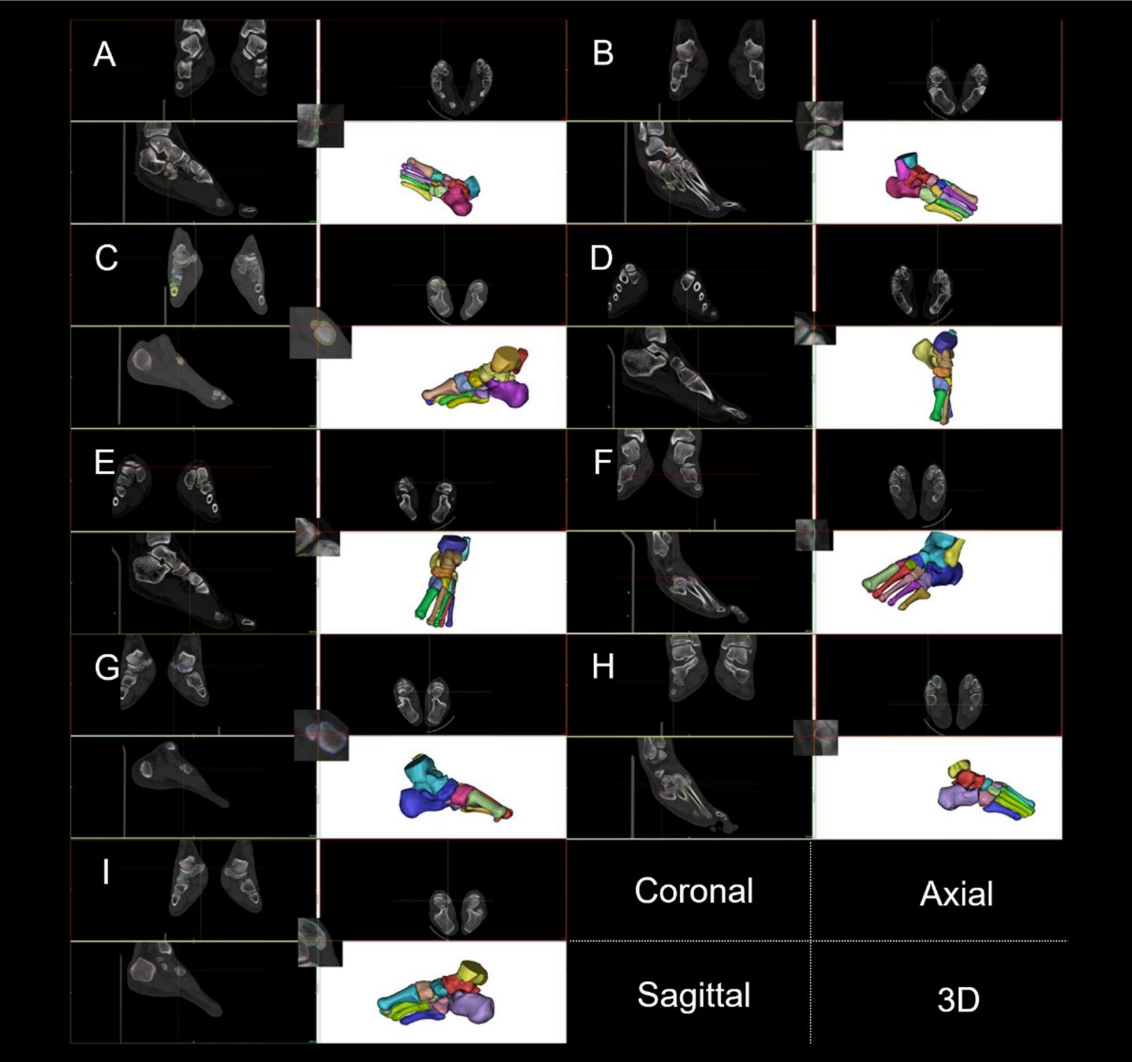
Fractures of participants’ cuboid, cuneiform, and navicular. **A** P2 right cuboid. **B** P2 right navicular. **C** P4 right navicular. **D** P7 left medial cuneiform. **E** P7 left navicular. **F** P10 left cuboid. **G** P10 left navicular. **H** P10 right cuboid. **I** P10 right navicular

**Fig. 8:**
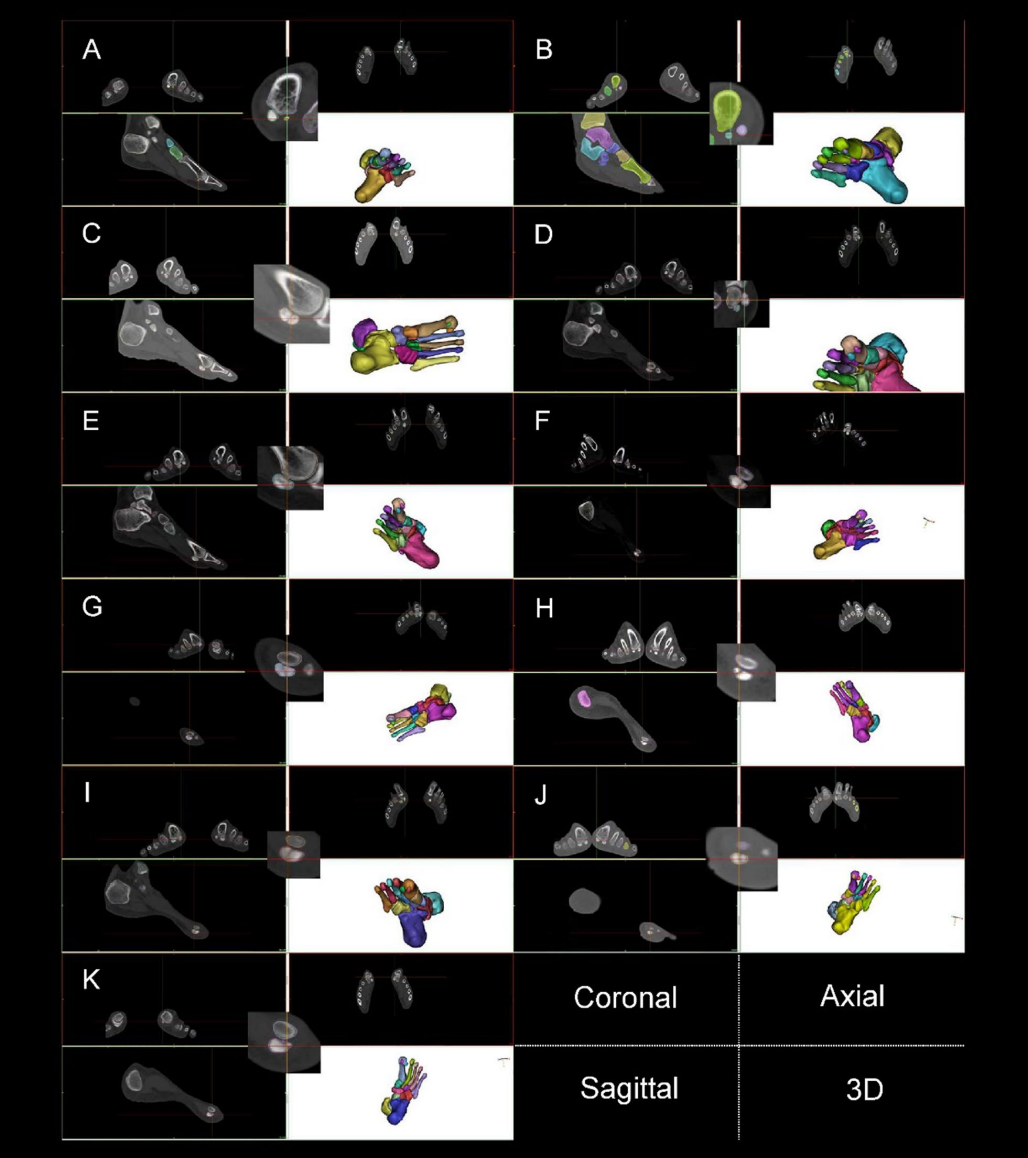
Fractures and ablation of participants’ sesamoid. **A** P1 left lateral sesamoid. **B** P1 right lateral sesamoid. **C** P2 left lateral sesamoid. **D** P2 right median sesamoid. **E** P2 right lateral sesamoid. **F** P3 left median sesamoid. **G** P3 right median sesamoid. **H** P5 right median sesamoid. **I** P6 right median sesamoid. **J** P8 left median sesamoid. **K** P9 left median sesamoid

In this investigation, we examined the CT images (Fig. 3) and 3D images (Fig. 4) of participants to obtain clinical information regarding foot and ankle SI. The reconstructed ankle structures were observed, and various bone deformities were revealed, including those from the tibia, fibula, talus, calcaneus, navicular, and first metatarsal. There were 51 cases in total, of which 7 had a flattened lateral sesamoid at the first metatarsal bone (i.e., the SG curvature diameter ≥ 100 mm). Within Mimics software, the 3D reconstructed tibia, fibula, tarsus, metatarsus, and sesamoid were combined with the reconstructed CT images at the axial, coronal, and sagittal positions to examine foot and ankle SI in participants (See Figs. 5, 6, 7, 8). Ankle fractures and ablation in Figs. 5, 6, 7, 8 were recorded. See Table 3 for details.

Figure 5 and Table 3 show 12 fractures at the tibia and fibula in 7 participants, including avulsion fractures at the anterior tibiofibular, interosseous tibiofibular, and superior anterior talofibular ligament attachment points. Figure 6 and Table 3 show 8 fractures at the talus in five participants, all at the lateral process of the talus. One participant developed bilateral fractures without osseous connections. Figure 7 and Table 3 show 5 navicular fractures from 4 participants, all at the spring ligament complex, plantar cuneocuboid ligament, and plantar calcaneocentral ligament attachment points [26]. In this study, bilateral navicular fractures occurred in one participant at the same time, which may be related to that participant’s techniques. In addition, three cuboid avulsion fractures were observed in two participants and one avulsion fracture at the naviculocuneiform ligament attachment point on the medial cuneiform bone was observed in one participant.

Figure 8 and Table 3 show 11 fractures and ablation at the sesamoid from 7 participants. The medial and lateral SGs of the first metatarsal bone affect the role of the flexor hallucis brevis and flexor hallucis longus muscles and determines the efficiency of the windlass mechanism. Table 4 shows the SG curvature diameters in Fig. 1.

**Table 4:** Participants’ curvature diameters of the first metatarsal grooves for sesamoids and their postures

	M-G		L-G	
	Ex. (°)	Di. (mm)	Ex. (°)	Di.(mm)
P1				
Left	0.5	18.15	0.0	29.10
Right	− 0.5	10.32	− 1.0	55.87
P2				
Left	− 1.0	4.43	0.0	Flat
Right	− 2.0	4.41	0.0	Flat
P3				
Left	− 1.5	1.60	1.0	16.48
Right	2.5	3.80	0.0	18.18
P4				
Left	− 1.0	7.09	1.0	4.86
Right	− 0.5	7.03	0.0	7.58
P5				
Left	0.0	8.25	1.5	27.69
Right	1.0	14.44	0.5	40.34
P6				
Left	− 2.0	5.40	− 0.5	7.83
Right	0.0	6.20	2.5	18.65
P7				
Left	0.5	9.59	0.0	Flat
Right	0.0	7.09	0.0	Flat
P8				
Left	0.5	34.29	2.0	23.16
Right	0.0	12.03	0.5	13.16
P9				
Left	− 0.5	83.06	0.0	Flat
Right	− 2.0	8.39	0.0	Flat
P10				
Left	− 2.0	7.32	1.5	31.28
Right	2.0	3.54	0.0	Flat

After the finite element models of the three fencers were processed in Ansys, the effects of the medial and lateral sesamoid and SGs on the efficiency of the first MTPJ were presented, i.e., the flexor pollicis brevis muscle and the cuboid and proximal phalanx were connected and passed through the SG of the first metatarsal bone. Therefore, the parallel SGs changed their attachment points. The non-parallel medial and lateral SGs increased the eccentric force while the parallel medial and lateral SGs decreased the eccentric force [20]. The Max-PS and Min-PS peak difference between the medial and lateral sesamoids and the TS difference (lateral minus medial) were recorded and analysed and the results were shown in Table 5.

**Table 5:** Max-PS, Min-PS and TS differences of the SG of the participants’ first metatarsals

Rotation (°)	D-value of Max-PS (mpa)			D-value of Min-PS (mpa)			D-value of TS (n)		
	P2	P3	P7	P2	P3	P7	P2	P3	P7
0	0.1584	0.1714	0.0036	0.3442	0.1584	0.0032	3.50	4.75	38.33
5	0.1599	0.1482	0.0089	0.3554	0.1599	0.0092	4.37	8.59	39.75
10	0.1619	0.1201	0.0120	0.3518	0.1619	0.0576	5.19	12.33	40.92
15	0.1598	0.1000	0.0179	0.3691	0.1598	0.0985	5.97	15.89	41.82
20	0.1591	0.0758	0.0206	0.3871	0.1591	0.0749	6.69	19.23	42.47
25	0.1625	0.0560	0.0216	0.3751	0.1625	0.0776	7.34	22.32	42.87
30	0.1555	0.0380	0.0252	0.3892	0.1555	0.0801	7.92	25.12	43.06
35	0.1505	0.0209	0.0291	0.3927	0.1505	0.0784	8.44	27.64	43.06
40	0.1465	− 0.0046	0.0320	0.3883	0.1465	0.0886	8.89	29.90	42.91
45	0.1405	− 0.0181	0.0332	0.3967	0.1405	0.0852	9.29	31.88	42.65
Average	0.1554	0.0708	0.0204	0.3749	0.1554	0.0653	6.76	19.77	41.78
CV	4.46%	85.72%	46.16%	4.78%	4.46%	47.76%	27.63%	44.33%	3.69%

*Key* Rotation: ankle joint angle in lunge. *Max-PS* the maximum principal stress peak difference at the medial and lateral sesamoid in the trail foot in lunge; *Min-PS* the minimum principal stress peak difference at the medial and lateral sesamoid in the trail foot in lunge. Total stress: the TS difference between the medial and lateral sesamoids in the trail foot in lunge

The stress distribution at the sole during lunging for three fencers were measured by Zebris FDM gait analysis system. The stress at the medial anterior foot was 93.3 ± 44.1 N, 260.7 ± 18.4 N, and 179.4 ± 32.6 N, respectively, which was close to the FEA results.

## Discussion

The aim of this investigation was to screen and diagnose asymptomatic ankle SI in fencers by using CT images and 3D reconstructions. Finite element models were generated to estimate the influence of each injury on the elite fencers’ loading conditions and to provide evidence as to whether the asymptomatic injury would increase the risk of serious injuries. An investigation of this nature therefore adds to the current literature base in fencing by providing important clinical information regarding injury evaluation and prediction of fencers.

Research in young athletes has shown that asymptomatic foot radionuclide bone imaging abnormalities and diffuse tibia abnormalities were common, and that asymptomatic lower limb abnormalities accounted for about 34% of injuries [27]. MRI observations of the tibia in 21 elite college long-distance runners shows signs of 43% of tibial stress reaction [28]. MRI findings showed a 77% (i.e., 30 out of 39) asymptomatic hockey players with pathological abnormalities in the hip or groin [29]. Imaging diagnosis revealed that the frequency of foot and ankle injuries in fencers is much higher than expected as almost all participants showed different levels of SI (Table 3). Caution should be paid to interpret the association between these findings and the future severe sports injuries [29].

Higher ankle joint angles in lunges make the ankle experience high-energy axial, rotational, and horizontal stress, which are not evenly distributed [4], leading to a high frequency of foot and ankle SI. Importantly, the anterior tibiofibular and interosseous tibiofibular ligament are part of the distal tibiofibular syndesmosis [30] and avulsion fracture decreases the stability of the distal tibia and fibula. The lateral process of the talus is not only the attachment point for the posterior talofibular ligament, but also a partial attachment point for the support belt of the flexor hallucis longus. The posterior talofibular and the talofibular ligament jointly stabilize the ankle joint [31]. Ligamentous laxity may further lead to ankle joint instability and increase the risk of sports injuries [32], such as tarsal fractures. Indeed, Table 3 and Fig. 1 showed that participants had 5 other tarsal fractures in addition to talus fractures. Although unable to determine the sequence of fractures, our observations confirm that decreased ankle joint stability increases the risk of fractures in other tarsi. The dorsiflexion of the trail foot and strike pattern of the trail foot in lunges [17] were shown to increase the risk of fractures at the lateral process of the talus. Fractures at the lateral process of talus were observed in both feet in 3 participants. In addition, 2 participants had bilateral avulsion fractures at the calcaneonavicular ligament and calcaneocuboid ligament attachment points. Fractures at the spring ligament complex, plantar cuneocuboid ligament, and plantar calcaneocentral ligament attachment points in the trail foot may be related to the dorsiflexion movement of the trail foot in lunges, while fractures at the lead foot may be related to the retraction movement in lunges [1].

Figures 5 and 8 as well as Table 4 show that structural fractures were present at the sesamoid of the left leg in 6 participants and of the lead foot in 2 participants. Among 20 feet, 9 sesamoid fractures and 7 flat lateral SGs were observed, resulting in SG and ridge ablation. The sesamoid and first metatarsal bone are the framework parts of the MTPJ. The morphology of the medial and lateral sesamoids and the SGs determines the contraction results of the flexor hallucis longus and flexor hallucis brevis muscles [33]. Therefore, sesamoid fractures, SG and ridge ablation weaken the windlass efficiency of the first MTPJ [31]. Sesamoid fractures at the trail foot may be associated with dorsiflexion movements of the trail foot whilst sesamoid fractures at the lead foot may be related to the retraction movements during the lunge [17]. The medial and lateral sesamoids are components of the first MTPJ, and thus essential to the function of MTPJ [34]. We found that since sesamoid had one of the highest injury incidences for fencers, it should be included as an occupational injury for the elite fencers.

By combining with imaging diagnosis results, P2 was found to have a flat lateral SG (Table 4). A flat SG lacks geometric constraints to the sesamoid, impairs the contraction of the flexor hallucis brevis muscles, reduces the efficiency of the propulsive force, and thus increases the risk of injury or lesion of the first MTFJ (e.g., hallux valgus). Therefore, the high principal stress of the medial SG caused a medial sesamoid fracture in P2 (Table 3). Typically, the risk of medial sesamoid fracture is higher, due to the strong impact on the MTPJ when the trail foot lunges. On the other hand, the increase of ankle joint angle in the sagittal plane enlarges TS difference between the medial and lateral SGs. These asymmetric changes to the Max-PS and TS increase the risk of avulsion fractures [35], so multiple ankle injuries are observed in the left foot of P2 (Table 3). The medial sesamoid fracture in P2 brings greater TS to the lateral SG, and the Max-PS and Min-PS of the medial and lateral sesamoids also becomes greater, generating great tension to the ligaments and muscles of the first MTPJ [35]. Tension generated by the dorsiflexed position of the foot and the limitation of ankle joint rotation increases the risk of foot varus, affecting the tibia and fibula by a torsional moment while pulling to the meniscus. Because the meniscus inside the knee joint capsule is thin in the middle and thick in the edge, when the knee joint extends backward, the thicker edge of the meniscus is in the knee joint cavity, reducing the range of motion in it, leading to unavoidable friction. So, foot varus can trigger injury to multiple ligaments (e.g., anterior talofibular ligament) and tendons (e.g., Achilles tendon) or even to the meniscus.

P3’s Max-PS of the medial and lateral sesamoids are very high, maybe resulting from his smallest curvature diameter of the medial and lateral sesamoids among the selected three participants. Also, his medial sesamoid groove leans to the lateral side (Table 4, − 1.5°). The medial sesamoid groove does not parallel much with the lateral one, considerably affecting the MTPJ angle to load force. During the propulsion phase of the lunge, power is generated by the ankle plantar flexors and knee/hip extensors of the trail leg.

Lunge can be successfully performed by stepping the fore foot and extending the rear foot, generating more hip flexion force [36]. Once the trail foot passes in front of the lead foot, the thrust-absorption cycle is repeated with a reversal of the power and of absorption of the lower limbs [37]. The overly high CV of P3 indicates a high risk of the above-mentioned injury in future training and competition, especially when using a lunge technique with a larger ankle angle. P7’s mean TS difference and CV between the medial and lateral sesamoids are kept at a stable level − 41.78 N and 3.69%, respectively (Table 5). When the ankle joint angle changes, the principal stress peak at the medial SG is usually higher than that at the lateral SG, while the TS value gives the opposite results, suggesting that the risk of stress fractures on the medial sesamoid increases when the rotation angle of the ankle joint is too wide in lunges.

Different types of MTPJ structures have different stress distribution characteristics, which were all affected by an increased ankle joint angle, leading to instability at the ankle itself [1]. These observations should attract attention from fencers because foot and ankle SI reduce the stability of the lower limb and might cause more injuries to the foot and ankle or even the knee and waist. There are many tactical and technical decisions that affect how the lunge is executed [18], but an excessively large trail foot angle will not optimally generate force or speed. Therefore, fencers should seek to reduce the rotation angle of the ankle joint at the trail foot in lunges as long as the quality of performance can be maintained.

A limitation of this study is that it is a cross-sectional analysis. Future longitudinal follow-up investigations concerning symptom development of these fencers should be conducted to prove the predictive function of the asymptomatic foot and ankle SI. Identifying the kinematic and kinetic characteristics of different asymptomatic ankle SI of participants may have potential in distinguishing asymptomatic ankle SI, so the symptom development of athletes can be monitored by routine biomechanical analyses.

This study utilized a simplified FEA whereby the performance of soft tissues was not considered, which may reduce the applicability of this model. For instance, we cannot estimate plantar pressure, nor the stress variation caused by different metatarsal bones’ geometric shape to the soft tissues. Also, our finite element model was designed based on single subject, even though the geometric model was normalized to assume bone structure to be the only difference, the results could not represent target population of different age, weight and bone mass characteristics.

## Conclusions

In this study, none of our participants had serious foot or ankle injury, but imaging analysis revealed different deformities. These asymptomatic foot and ankle SI may affect the work efficiency of muscles or the stability of the foot and ankle. Furthermore, FEA demonstrated that damaged structures of the MTPJ changed the mechanical environment of lunges and might lead to avulsion fractures and stress fracture, increased ankle joint angle in the trail foot may further increase these risks. We propose that asymptomatic foot and ankle SI can lead to more frequent or more severe lower limb injury in athletes, where coaches and athletes should be alert. Fencers can consciously control their rotation angle of the trail foot in lunges to decrease the risk of MTPJ injury. Asymptomatic foot and ankle SI were occupational pathologies to the fencers, and the accumulation of such SI may change the biomechanical features of the fencers’ foot and ankle. FEA results indicate that more severe injuries might follow with a higher incidence rate. How to accurately diagnose SI should be a scientific problem to be solved by more follow-up clinical studies. Regular monitoring can successfully evaluate and predict the injury risk and that foot and ankle SI may be prevented with the help of biomechanical principles and instruments.

## Data Availability

The datasets used and/or analysed during the current study available from the corresponding author on reasonable request.

## Abbreviations

SI: Structural injuries
FEA: Finite element analysis
MTPJ: First metatarsophalangeal joint
SG: Sesamoid groove
Max-PS: Maximum principal stress
Min-PS: Minimum principal stress
TS: Total stress
CV: Coefficient of variation

## References

[1] Chen TL-W, Wong DW-C, Wang Y, Ren S, Yan F, Zhang M. Biomechanics of fencing sport: a scoping review. PLoS ONE. 2017;12(2):e0171578. 10.1371/journal.pone.0171578 PMC5302478 28187164

[2] Harmer PA (2008). Incidence and characteristics of time-loss injuries in competitive fencing: a prospective, 5-year study of national competitions. Clin J Sports Med.

[3] Wild A, Jaeger M, Poehl C, Werner A, Raab P, Krauspe R (2001). Morbidity profile of high-performance fencers. Sportverletz Sportsc.

[4] Zaricznyj B (1977). Avulsion fracture of the tibial eminence: treatment by open reduction and pinning. J Bone Jt Surg Am.

[5] Giombini A, Dragoni S, Di Cesare A, Di Cesare M, Del Buono A, Maffulli N (2013). Asymptomatic Achilles, patellar, and quadriceps tendinopathy: a longitudinal clinical and ultrasonographic study in elite fencers. Scand J Med Sci Sports.

[6] Walrod B, Turner W, Hartz C (2019). A prospective cohort study of collegiate fencing injuries. Curr Sport Med Rep.

[7] Harmer PA (2019). Epidemiology of time-loss injuries in international fencing: a prospective, 5-year analysis of Federation Internationale d’Escrime competitions. Br J Sports Med.

[8] Greaser MC (2016). Foot and ankle stress fractures in athletes. Orthop Clin N Am.

[9] Hussey PS, Timbie JW, Burgette LF, Wenger NS, Nyweide DJ, Kahn KL (2015). Appropriateness of advanced diagnostic imaging ordering before and after implementation of clinical decision support systems. JAMA-J Am Med Assoc.

[10] Fan Y, Luo L, Djuric M, Li Z, Antonijevic D, Milenkovic P, Sun Y, Li R, Fan Y (2017). Extracting cross-sectional clinical images based on their principal axes of inertia. Scanning.

[11] Paul SK, Sivarasu S, Mathew L (2012). Customized foot orthosis development by 3D reconstruction of the CT images. Engineering.

[12] Gu YD, Ren XJ, Li JS, Lake MJ, Zhang QY, Zeng YJ (2010). Computer simulation of stress distribution in the metatarsals at different inversion landing angles using the finite element method. Int Orthop.

[13] Li S, Zhang Y, Gu Y (2017). Stress distribution of metatarsals during forefoot strike versus rearfoot strike: a finite element study. Comput Biol Med.

[14] Kristen K-H, Berger K, Berger C (2005). The first metatarsal bone under loading conditions: a finite element analysis. Foot Ankle Clin.

[15] García-Aznar JM, Bayod J, Rosas A, et al. Load transfer mechanism for different metatarsal geometries: a finite element study. J Biomech Eng. 2008;131(2): 021011. 10.1115/1.3005174 19102570

[16] Aquili A, Tancredi V, Triossi T, De Sanctis D, Padua E, D’Arcangelo G, Melchiorri G (2013). Performance analysis in saber. J Strength Cond Res.

[17] Sinclair J, Bottoms L (2013). Gender differences in the kinetics and lower extremity kinematics of the fencing lunge. Int J Perform Anal Sport.

[18] Gresham-Fiegel CN, House PD, Zupan MF (2013). The effect of nonleading foot placement on power and velocity in the fencing lunge. J Strength Cond Res.

[19] Li R, Fan Y, Liu Y, Antonijevic Ð, Li Z, Djuric M, Fan Y (2019). Homo naledi did not have flat foot. Homo.

[20] Fan Y, Antonijevic D, Antic S, Li R, Liu Y, Li Z, Djuric M, Fan Y (2019). Reconstructing the first metatarsophalangeal joint of homo naledi. Front Bioeng Biotechnol.

[21] Yu G, Fan Y, Fan Y, Li R, Liu Y, Antonijevic D, Milovanovic P, Zhang B, Li Z, Djuric M, Fan Y (2020). The role of footwear in the pathogenesis of hallux valgus: a proof-of-concept finite element analysis in recent humans and homo naledi. Front Bioeng Biotechnol.

[22] Gutierrez-Davila M, Rojas FJ, Antonio R, Navarro E (2013). Response timing in the lunge and target change in elite versus medium-level fencers. Eur J Sport Sci.

[23] Yemineni BC, Mahendra J, Nasina J, Mahendra L, Shivasubramanian L, Perika SB. Evaluation of maximum principal stress, von mises stress, and deformation on surrounding mandibular bone during insertion of an implant: a three-dimensional finite element study. Cureus. 2017;12(7):e9430. 10.7759/cureus.9430 PMC7450897 32864255

[24] Owen DRJ, Prakash A, Zienkiewicz OC (1974). Finite element analysis of non-linear composite materials by use of overlay systems. Comput Struct.

[25] Liu Y, Li R, Fan Y, Antonijevic D, Milenkovic P, Li Z, Djuric M, Fan Y. The influence of anisotropic voxel caused by field of view setting on the accuracy of three-dimensional reconstruction of bone geometric models. AIP Adv. 2018;8(8):085111.

[26] Krahenbuhl N, Horn-Lang T, Hintermann B, Knupp M (2017). The subtalar joint: a complex mechanism. EFORT Open Rev.

[27] Drubach LA, Connolly LP, D’Hemecourt PA (2001). Assessment of the clinical significance of asymptomatic lower extremity uptake abnormality in young athletes. J Nucl Med.

[28] Bergman AG, Fredericson M, Ho C (2004). Asymptomatic tibial stress reactions: MRI detection and clinical follow-up in distance runners. Am J Roentgenol.

[29] Silvis ML, Mosher TJ, Smetana BS (2011). High prevalence of pelvic and hip magnetic resonance imaging findings in asymptomatic collegiate and professional hockey players. Am J Sports Med.

[30] Yuen CP, Lui TH (2017). Distal tibiofibular syndesmosis: anatomy, biomechanics, injury and management. Open Orthop J.

[31] Szaro P, Ghali Gataa K, Polaczek M, Ciszek B (2020). The flexor retinaculum connects the surrounding structures into the medial ankle complex. Appl Sci.

[32] Hollis JM, Blasier RD, Flahiff CM. Simulated lateral ankle ligamentous injury. Change in ankle stability. Am J Sports Med. 1995;23(6):672–7. 10.1177/036354659502300606 8600732

[33] Zifchock R, Parker R, Wan W, Neary M, Song J, Hillstrom H (2019). The relationship between foot arch flexibility and medial-lateral ground reaction force distribution. Gait Posture.

[34] Welck MJ, Singh D, Cullen N, Goldberg A (2018). Evaluation of the 1st metatarso-sesamoid joint using standing CT—the Stanmore classification. Foot Ankle Surg.

[35] Harris E, Moroney P, Tourne Y (2017). Arthrodesis of the first metatarsophalangeal joint—a biomechanical comparison of four fixation techniques. Foot Ankle Surg.

[36] Bottoms L, Greenhalgh A, Sinclair J (2013). Kinematic determinants of weapon velocity during the fencing lunge in experienced epee fencers. Acta Bioeng Biomech.

[37] Dauty M, Gross R, Leboeuf F, Trossaert M (2015). Comparison of total ankle replacement and ankle arthrodesis in patients with haemophilia using gait analysis: two case reports. BMC Res Notes.

